# Leukocyte extract reduces HIV replication and modulates cellular factors involved in HIV infection: therapeutic meant

**DOI:** 10.1186/1758-2652-13-S4-P226

**Published:** 2010-11-08

**Authors:** C Fernandez-Ortega, D Casillas, M Dubed, L Navea, A Ramirez, L Lopez, T Paneque, Y Reinoso

**Affiliations:** 1 Center for Genetic Engineering and Biotechnology, Cell Biology, Havana, Cuba; 2 AIDS Research Institute, Havana, Cuba; 3 Center for Genetic Engineering and Biotechnology, Havana, Cuba

## Background

The development of antiretroviral therapies to combat human immunodeficiency virus (HIV) infection has resulted in a decrease in morbidity and mortality associated with the acquired immunodeficiency syndrome (AIDS). Despite these therapeutic advances, problems of drug resistance, latent viral reservoirs, and drug induced toxic effects that compromise effective viral control point to the need for new classes of anti-HIV drugs with different modes of action. Dialyzable Leukocyte Extract (DLE) is a low molecular weight dialyzable material obtained from human leukocytes. A clinical trial of six years of follow-up was carried out using a DLE preparation in asymptomatic HIV patients. Twenty-eight percent of the untreated individual showed disease progression, while only progressed to AIDS 7% of DLE-treated patients. These results indicate that DLE delays disease progression. However, the molecular basis supporting this effect remained unknown.

## Purpose of the study

To demonstrate anti-HIV activity in DLE and show DLE modulation on cellular factors involved in HIV replication.

## Methods

Using an in vitro infection model on MT4 cell line we study the effect of DLE on HIV replication. We study the effect of DLE on important cellular factors like NFkB, Sp1 and TNF in MT4 cells or peripheral blood mononuclear cells.

## Summary of results

**Figure 1:**
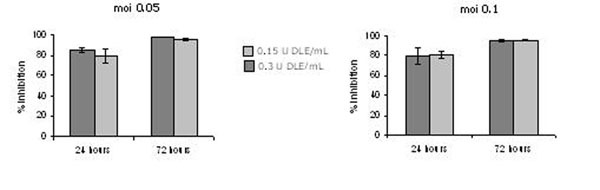


DLE shows a significant inhibitory effect on HIV replication ranged from 80-90% according to the viral challenge (figure 1. In addition, others results shown DLE modulation of important endogenous factors involved in HIV immunopathogenesis like TNFa and transcription factors NF©§B and Sp1.

## Conclusions

DLE effect on cellular factors involved in HIV replication correlates with DLE inhibitory effect on HIV in vitro replication. The inhibition of HIV replication observed with DLE treatment could be mediated by inhibition of transcription factors that may promote replication of HIV. Also, it could be mediated or potentiated by modulation TNF and others endogenous factors involved in HIV replication. These finding could support the use of DLE on HIV patients.

